# Stemness Analysis Uncovers That The Peroxisome Proliferator-Activated Receptor Signaling Pathway Can Mediate Fatty Acid Homeostasis In Sorafenib-Resistant Hepatocellular Carcinoma Cells

**DOI:** 10.3389/fonc.2022.912694

**Published:** 2022-07-22

**Authors:** Tingze Feng, Tianzhi Wu, Yanxia Zhang, Lang Zhou, Shanshan Liu, Lin Li, Ming Li, Erqiang Hu, Qianwen Wang, Xiaocong Fu, Li Zhan, Zijing Xie, Wenqin Xie, Xianying Huang, Xuan Shang, Guangchuang Yu

**Affiliations:** ^1^ Department of Bioinformatics, School of Basic Medical Sciences, Southern Medical University, Guangzhou, China; ^2^ Department of Medical Genetics, School of Basic Medical Sciences, Southern Medical University, Guangzhou, China; ^3^ Country Guangdong Provincial Key Laboratory of Viral Hepatitis Research, Hepatology Unit and Department of Infectious Diseases, Nanfang Hospital, Southern Medical University, Guangzhou, China; ^4^ Division of Vascular and Interventional Radiology, Department of General Surgery, Nanfang Hospital, Southern Medical University, Guangzhou, China

**Keywords:** hepatocellular carcinoma, sorafenib resistance, PPAR signaling pathway, stemness index, prognosis

## Abstract

Hepatocellular carcinoma (HCC) stem cells are regarded as an important part of individualized HCC treatment and sorafenib resistance. However, there is lacking systematic assessment of stem-like indices and associations with a response of sorafenib in HCC. Our study thus aimed to evaluate the status of tumor dedifferentiation for HCC and further identify the regulatory mechanisms under the condition of resistance to sorafenib. Datasets of HCC, including messenger RNAs (mRNAs) expression, somatic mutation, and clinical information were collected. The mRNA expression-based stemness index (mRNAsi), which can represent degrees of dedifferentiation of HCC samples, was calculated to predict drug response of sorafenib therapy and prognosis. Next, unsupervised cluster analysis was conducted to distinguish mRNAsi-based subgroups, and gene/geneset functional enrichment analysis was employed to identify key sorafenib resistance-related pathways. In addition, we analyzed and confirmed the regulation of key genes discovered in this study by combining other omics data. Finally, Luciferase reporter assays were performed to validate their regulation. Our study demonstrated that the stemness index obtained from transcriptomic is a promising biomarker to predict the response of sorafenib therapy and the prognosis in HCC. We revealed the peroxisome proliferator-activated receptor signaling pathway (the PPAR signaling pathway), related to fatty acid biosynthesis, that was a potential sorafenib resistance pathway that had not been reported before. By analyzing the core regulatory genes of the PPAR signaling pathway, we identified four candidate target genes, *retinoid X receptor beta* (*RXRB*), *nuclear receptor subfamily 1 group H member 3* (*NR1H3*), *cytochrome P450 family 8 subfamily B member 1* (*CYP8B1*) and *stearoyl-CoA desaturase* (*SCD*), as a signature to distinguish the response of sorafenib. We proposed and validated that the *RXRB* and *NR1H3* could directly regulate *NR1H3* and *SCD*, respectively. Our results suggest that the combined use of *SCD* inhibitors and sorafenib may be a promising therapeutic approach.

## Introduction

Primary liver cancer (PLC) is the fourth most common cause of cancer-related deaths worldwide and the sixth-most common cancer in the world, according to data provided by the World Health Organization (WHO) (1–3). HCC is the most common form of liver cancer and accounts for approximately 80% of all cases of PLC (4). Numerous patients were first diagnosed with advanced-stage HCC (5). Sorafenib, which is administered only as a first-line chemotherapeutic agent in advanced HCC patients, is the most prevalent oral small-molecule multi-kinase inhibitor and has been in use for over a decade (5, 6). Sorafenib can inhibit tumor cell proliferation and angiogenesis, thereby inducing cancer cell apoptosis, which not only blocks the Ras/MEK/ERK-mediated cell signaling pathway but also blocks tumor angiogenesis, by inhibiting kinases such as vascular endothelial growth factor receptor (VEGFR) and platelet-derived growth factor receptor (PDGFR) (1, 6). However, some HCC patients exhibited congenital resistance to sorafenib or acquired resistance after treatment (6, 7). Only a few patients with HCC exhibited extended survival after receiving sorafenib treatment (8). Therefore, we need to develop a method for predicting the response of HCC patients to sorafenib, to facilitate the precise treatment of advanced HCC patients. Importantly, we need to identify the primary and additional mechanisms of acquired sorafenib resistance.

Stemness was defined as the ability of cells to self-renewal and interact with their environment to maintain a balance between quiescence, proliferation, and regeneration (9, 10). Normal adult stem cells exhibit stemness when involved in tissue homeostasis, whereas cancer stem cells (CSCs) display stemness when involved in malignant growth (10, 11). Moreover, it had been proven that non-CSCs can dedifferentiate into CSCs by the stimuli of the tumor microenvironment (11, 12). Cancer progression necessitates the gradual loss of a differentiated phenotype and restoration of progenitor and stem cell-like features (11, 13, 14). Both patient prognosis and drug response are likely to be related to the state of tumor cells (15). Evidence has shown that an assessment of tumor stemness can reflect tumor status, and liver cancer stem cells can mediate tumor growth and sorafenib resistance development (7, 16). Undifferentiated HCC is more likely to result in tumor metastasis, disease recurrence, poor prognosis, and drug resistance (7, 17). However, there is a lack of systematic studies examining the relationship between sorafenib resistance and the HCC stemness index. In recent years, despite several efforts to identify potential biomarkers in HCC patients’ prognosis, only a few have focused on drug response (18). Numerous HCC risk signatures were identified by gene expression for predicting HCC patient prognosis. Whereas these signatures were typically validated using only a single dataset or were not externally validated (19), resulting in unreliable clinical outcome prediction. There is still an urgent need for reliable and robust markers that can be used for predicting the prognosis of different HCC cohorts and the effect of drug therapy, which are also of great value for the precise treatment of patients. Several studies have shown that the stemness index is effective for the prediction of prognosis and drug resistance in multiple malignancies (20–23). Here, we aimed to explore the regulatory mechanism under sorafenib-resistant conditions using the stemness index.

In this study, the cancer stemness was assessed by extracting sets of transcriptomic features (mRNAsi), using the one-class logistic regression (OCLR) machine-learning algorithm, which was proposed in a recent study (22). We systematically analyzed HCC stem-like indices using a total of 7 independent HCC cohorts and the OCLR algorithm. We identified mRNAsi-related subgroups that can distinguish between different responses to sorafenib treatment and evaluated the prognostic significance of mRNAsi in several datasets. To our knowledge, this was the first attempt to use the tumor stemness index to explore the potential mechanisms of sorafenib drug resistance development. Moreover, we identified that four genes, which were found to be involved in the PPAR signaling pathway, might play a role in sorafenib resistance development. The signature for predicting sorafenib treatment effectiveness has been extracted. We additionally discussed the regulation of SCD and its upstream genes in the PPAR signaling pathway by combining other omics data such as somatic mutations of key genes, transcription factor binding site for key genes, and methylation level of the SCD promoter. And luciferase reporter assays were performed to validate regulations of key genes.

## Materials and Methods

### Patient Cohorts and Clinical Data

Publicly available data regarding HCC cohorts were systematically screened and checked, and matched with individual clinical annotations. In total, we obtained seven HCC cohorts, involving a total of 991 samples; of these, five cohorts were used for the survival study (TCGA-HCC, ICGC-JP, GSE14520 (24), GSE76427 (25), GSE116174), and the other two were used for a sorafenib drug response study (GSE109211 (26) cohort and GSE143477 (27) cohort). The expression profiles of the TCGA cohort were obtained through a data portal (https://xenabrowser.net/) (28), along with both somatic mutation data and clinical data of tumor samples. Besides, the GSE14520, GSE76427, GSE116174, GSE109211, and GSE143477 cohorts were obtained from the Gene Expression Omnibus (http://www.ncbi.nlm.nih.gov/geo/). Sixty-seven samples treated with sorafenib were contained in the GSE109211. The sorafenib samples of GSE109211 were divided into “responder” (n=21) and “non-responder” (n=46) groups in terms of recurrence-free survival (RFS). Compared with the responder group in GSE109211, sorafenib non-responders were defined as patients in whom sorafenib had no effect (sorafenib resistance). GSE143477 contains three sorafenib-resistant samples and three sorafenib-sensitive samples. The expression profiles and clinical information regarding the ICGC-JP cohort were downloaded from the ICGC Data Portal (https://dcc.icgc.org/). Three methods were adopted to collect clinical information: 1) Information was downloaded from the database if the authors had uploaded it; 2) Information was extracted from the original literature; and 3) It was obtained from the corresponding authors if necessary. All the information regarding these cohorts has been summarized in **Table 1**.

**Table 1:** Information regarding the HCC cohorts used in this study.

Cohort Names	Sample Size	Direction of Analysis	PMID	Link
TCGA-HCC	330	Prognosis	NA	https://xenabrowser.net/
ICGC-JP	229	Prognosis	NA	https://dcc.icgc.org/releases/release_28/Projects/LIRI-JP
GSE14520		Prognosis	21159642	
GSE76427		Prognosis	29117471	
GSE116174	359	Prognosis	NA	https://www.ncbi.nlm.nih.gov/geo/query/
GSE109211	67	Sorafenib response	30108162
GSE143477	6	Sorafenib response	32554246	

### Calculation of mRNAsi for HCC

**Figure 1:**
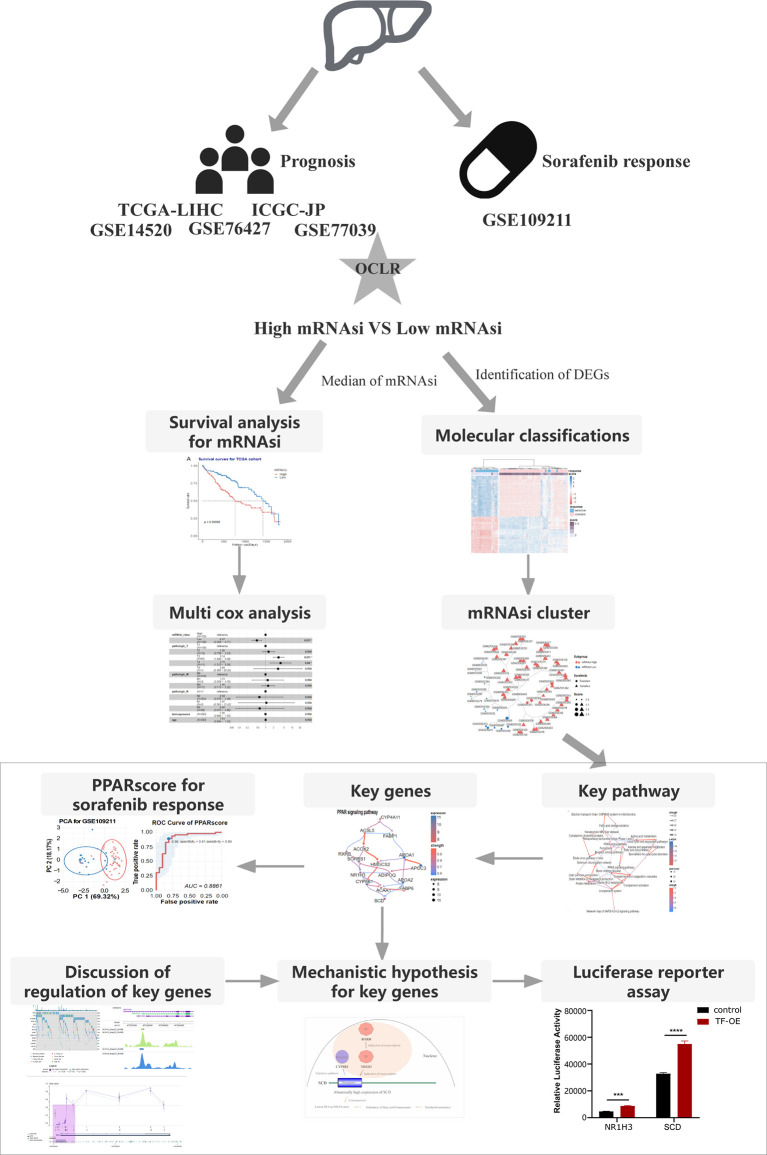
The flowchart demonstrates the analytical process of calculating HCC stemness and its association with the response to sorafenib treatment and HCC patient prognosis.

We collected samples from seven HCC cohorts, and derived their mRNA expression data and corresponding clinical information (survival or response to sorafenib), to characterize the mRNA stemness features of HCC patients, as demonstrated in the flowchart (**Figure 1**). We used an OCLR model based on the Progenitor Cell Biology Consortium (PCBC) embryonic stem cell data (29), to characterize the stemness signature. We collected 229 stem cell samples with 13013 protein-coding genes for use in the training dataset and assessed the stemness weight of each gene using the R package, *gelnet* (version 1.2.1) (30). The stemness weight of each gene has been shown in **Table S1**. These values were then applied to characterize the stemness features for each patient in a total of 6 HCC cohorts and obtain information regarding their mRNAsi. The mRNAsi, which range from 0 to 1, could serve as an indicator for assessing the degree of dedifferentiation of tumor samples.

We selected tumor samples from the TCGA-HCC cohort for inclusion in the survival training set and calculated the mRNAsi for the training set. We further classified the HCC patients in the TCGA-HCC cohort into the high-mRNAsi and low-mRNAsi groups, based on a median mRNAsi value of 0.55.

To verify the hypothesis that the lower the tumor differentiation, the higher the malignancy, and the worse the patient prognosis, we calculated the mRNAsi for five HCC cohorts. Tumor samples in the TCGA-HCC cohort were selected for inclusion in the training set and classified into the high-mRNAsi and low-mRNAsi groups, based on a median mRNAsi value of 0.55. Kaplan-Meier analysis (K-M analysis) was performed for the two groups. We validated the mRNAsi cutoff for prognosis prediction for the ICGC cohort and three GEO cohorts. In each cohort, tumor samples were divided into the high and low groups based on the mRNAsi cutoff value of 0.55. The validation of the prognostic significance of mRNAsi was also performed based on K-M analysis. R packages such as *survival* and *survminer (* 31–33) were used for performing K-M analysis and log-rank tests in all cohorts.

### Identification of the Relationship Between mRNAsi Subgroups and Sorafenib Response

After testing the hypothesis that mRNAsi can predict prognosis in HCC patients, we further hypothesized that mRNAsi may be associated with drug treatment sensitivity based on the activity of multiple inhibitors that were shown to be highly correlated with cancer stemness index mRNAsi (22). First, we identified differentially expressed genes (DEGs) between the high and low mRNAsi subgroups in the GSE109211 cohort. Sixty-seven samples were divided into two segments based on the median mRNAsi in the sorafenib cohort (median = 0.2513743). Additionally, 845 DEGs were analyzed among two segments using R package *limma (* 34)(| log2foldchange| > 1.5, adjusted p-value < 0.01).

Then, hierarchical clustering was performed based on DEGs using the R package *ConsensusClusterPlus (* 35), which used an algorithm to determine the cluster count and membership during unsupervised analysis. The process has repeated a total of 1000 times, to ensure the stability of the classification process; these samples were clustered into two groups based on the estimated number of clusters. The relationship between mRNAsi subgroups and the response to sorafenib was assessed and visualized using the R package *ggstatplot (* 36).

### Identification of Hub Pathways and Genes Involved in Sorafenib Resistance

After analyzing the relationship between mRNAsi subgroups and drug resistance, we further explored and evaluated the functional mechanisms that the DEGs between two subgroups might participate in, to identify molecular changes at the pathway level. WikiPathway enrichment analysis and visualization were performed *via clusterProfiler (* 37) and *enrichplot* packages. For focused critical pathways, the key regulatory genes involved also need to be analyzed. *CBNplot (* 38), which exhibited a Bayesian network inference approach, was employed to explore molecular regulatory relationships. We set the parameter *R* (the number of bootstraps) to 10000 to ensure that the gene or pathway regulatory network can be stably inferred.

### Generation and Validation of a PPAR-Related Signature for Sorafenib Resistance

We additionally examined whether the four hub genes identified by *CBNplot* could be used to distinguish the response to sorafenib. Principal component analysis (PCA) and visualization were performed *via FactomineR (* 39), *ggplot2*, and *ggstatplot (* 36). To assess the possibility of resistance to sorafenib, we used the first 2 principal components (number of dimensions: 2) to construct a PPAR-relevant gene signature. The signature scores contained the coordinates of samples in the first 2 principal components (PCs), which indicate the correlation between a sample and two principal components. The use of this method can enable the score to focus on the set with the largest block of correlated (or non-correlated) genes in the set. We then defined the PPAR-related signature score using a method similar to that used in Zhang’s study (40–42). *PPARscore* was defined as the risk score of sorafenib and was evaluated by adding the values for *Dimi1* and *Dimi2*. *Dimi1* was defined as the coordinate on PC1 of sample *i*. *Dimi2* was defined as the coordinate on PC2 of sample *i*. The formula used is as follows:

***PPARscore = Dimi1 + Dimi2***

Sixty-seven samples were divided into two segments according to the best threshold of *PPARscore*, which was the point closest to the upper left corner in the Receiver-operating characteristic (ROC) curve. Patients with PCA scores greater than the *PPARscore* cutoff (cutoff = -0.56) had a higher likelihood of developing sorafenib resistance. The *PPARscore* and its cutoff were validated in another sorafenib cohort, i.e., GSE143477.

### Analysis of Somatic Mutations for Hub Genes

We checked the mutation data of hub genes in the TCGA-HCC cohort, to examine whether the hub genes were affected by genomic alterations. We downloaded somatic variants in the mutation annotation format (MAF) and visualized the files. We compared the frequencies of somatic mutations in the top 10 mutational genes and key genes in the PPAR signaling pathway. The maftools R package was adopted for analysis (43).

### Visualization of Transcription Factor Binding Site for Key Genes

Cistrome Data Browser, a resource of human cis-regulatory data derived from Chromatin immunoprecipitation followed by sequencing (ChIP-seq). ChIP-seq profiling assays provide the genome-wide locations of transcription factor (TF) binding sites. We queried the potential binding transcript factors for specific genes in the Cistrome Data Browser (44, 45). Two *RXRB* ChIP-seq samples were used to analyze the binding of *RXRB* to the *NR1H3* promoter (ENCODE Project Consortium et al.) (46). In addition, four *NR1H3* ChIP-seq samples were used to analyze the binding of *NR1H3* to the *SCD* promoter (Savic D. et al.) (47).

### Luciferase Reporter Assay

To examine the effect of *RXRB* on *NR1H3* and *NR1H3* on *SCD* transcriptional activity, we constructed pGL4.18 vectors composed of *NR1H3* or *SCD* promoters. Empty pcDNA3.1 plasmid, pcDNA3.1-*RXRB*, or pcDNA3.1-*NR1H3* plasmid was co-transfected with pGL4.18-promoter vectors and phRL-TK plasmids using Lipofectamine 2000 in MHCC-97h cells. MHCC-97h cells were then harvested and luciferase activity was analyzed by using Dual-Luciferase^®^ Reporter Assay System kit (Promega). In order to compare the transfection efficiency, the firefly luciferase values were revised by the corresponding Renilla luciferase values.

### Visualization of Promoter DNA Methylation

We examined whether the expression levels of key genes related to the response to sorafenib were affected by methylation. We determined and visualized the methylation status of the SCD promoter using MEXPRESS (48), which is a web tool for generating fast queries and visualizing methylation data for the TCGA-HCC cohort.

### Statistical Analysis

Univariate survival analysis was performed *via* K-M survival analysis and the log-rank test. Correlation coefficients were assessed *via* Spearman analysis. Analyses of differentially expressed genes were performed based on the *limma* package, and K-M analysis was performed using the *survival* package and *survminer* package. Gene functional enrichment analysis was conducted *via clusterProfiler*. The mRNAsi-related subgroups were visualized using *ggtree*, *via* the generation of gene clustering trees (49). ROC curve was performed, and the area under the ROC curve was used to assess the predictive performance of *PPARscore* using the R package *pROC*. Different expressions between two groups (sorafenib-sensitive or sorafenib-resistant) were assessed using the Wilcoxon Rank Sum Test and P values adjusted by the hommel method. All statistical analyses were performed using R (Version 4.0.2), and the statistical significance was defined based on whether P < 0.05 or P < 0.01.

## Results

### mRNAsi Is Significantly Correlated With the Response to Sorafenib

**Figure 2:**
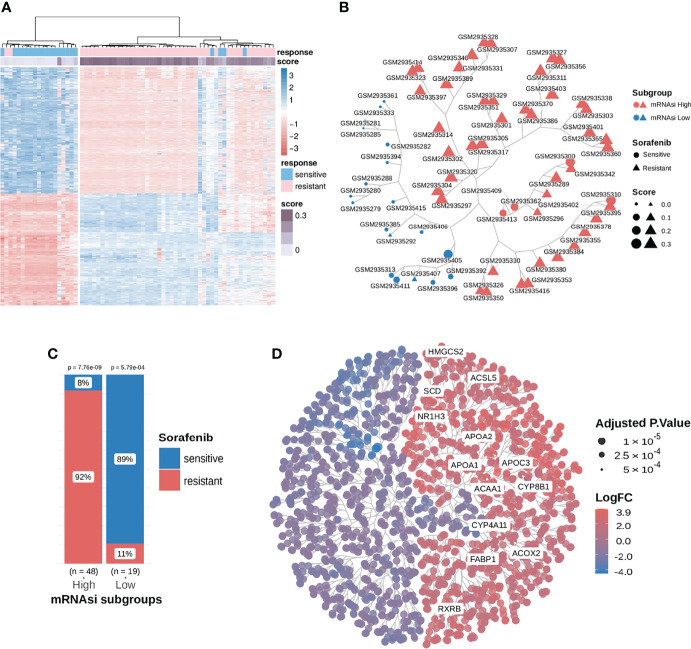
Consensus clustering facilitated the identification of distinct mRNAsi-related clusters associated with different responses to sorafenib treatment; samples in the mRNAsi-high cluster were resistant to sorafenib and exhibited higher stemness proportions. **(A)** We extracted and compared 845 DEGs using subgroup classification in the sorafenib cohort GSE109211. **(B)** Tree-based visualization of the two mRNAsi subgroups. **(C)** The proportion of different responses to sorafenib (responder or non-responder) in the two mRNAsi subgroups with statistical significance. **(D)** Tree cluster of 1853 differentially expressed genes. Genes in the PPAR signaling pathway have been highlighted. See also **Figure S1**.

The mRNAsi value was calculated as Spearman’s correlation between the weight vectors of the stemness signature using a *gelnet* trained OCLR model, based on the stem cell data (29, 30) and mRNA expression data for each of the HCC samples. Its value ranges between 0 to 1; a higher mRNAsi represents a lower level of differentiation in a sample, signaling drug resistance (16, 17). We explored mRNAsi subgroups to distinguish the response to sorafenib therapy. First, the mRNAsi scores of samples in the sorafenib cohort GSE109211 were calculated, and the samples were classified into the high and low subgroups using the median value of mRNAsi (0.2513743). Then, differential expression analysis was performed. Finally, 845 DEGs were identified using the screening criteria (|logFC| > 1.5, adj.P-value < 0.01) (**Figure 2A**). We could utilize those DEGs to cluster for identifying mRNAsi subgroups.

To investigate the relationship between DEGs, mRNAsi, and the response to sorafenib, we implemented a consensus clustering analysis for 67 patients from the GSE109211 cohort based on the expression pattern of 845 DEGs. The results revealed that there were two distinct patient clusters based on changes in the Cumulative Distribution Function (CDF) area and consensus matrix (**Figure S1**). As shown in **Figure 2B** and **Figure 2C**, the responses to sorafenib were notably different among the two subgroups. We termed two clustered subgroups as the mRNAsi-high subgroup and the mRNAsi-low subgroup. Only 8% of patients in the mRNAsi-high subgroup were sensitive to sorafenib, while 89% of patients in the mRNAsi-low subgroup exhibited a response to the therapy. The difference in the responses to sorafenib in the two mRNAsi-related subgroups was statistically significant (Test of proportion for mRNAsi-high subgroup p-value = 7.76e-9; Test of proportion for mRNAsi-low subgroup p-value = 5.79e-4). These results demonstrated that our mRNAsi-related subgroups could distinguish the drug response to sorafenib therapy in HCC patients.

### The PPAR Signaling Pathway Is the Key Pathway for Sorafenib Resistance

**Figure 3:**
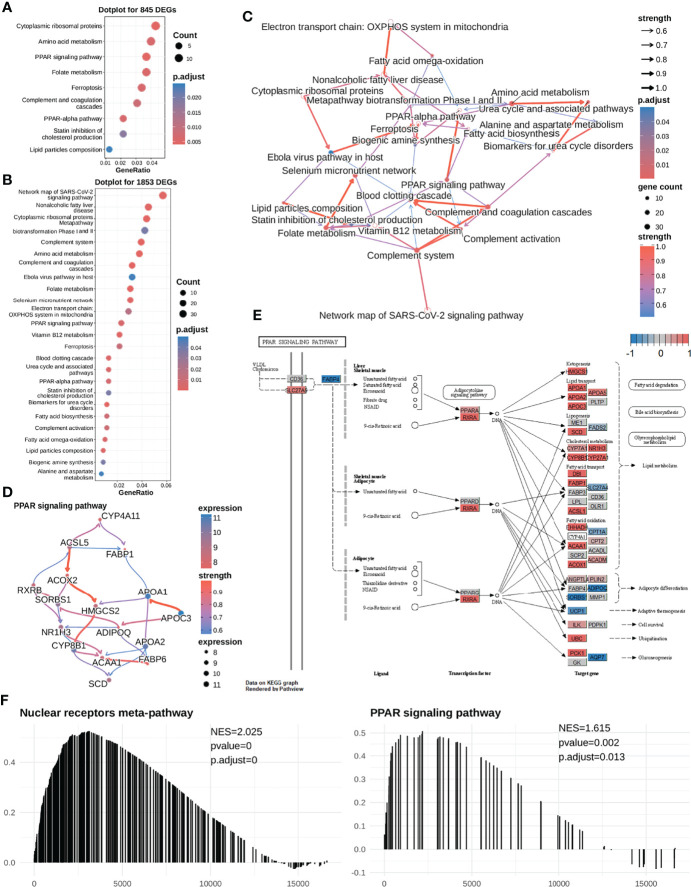
The high mRNAsi subgroup (sorafenib resistant) exhibits higher expression levels of most genes in the PPAR signaling pathway in HCC cells. **(A)** We first selected 845 DEGs for performing functional analysis and found that these DEGs were significantly enriched in the PPAR signaling pathway, cytoplasmic ribosomal proteins, folate metabolism, etc. **(B)** Next, we selected 1853 DEGS for performing enrichment analysis and found that the DEGs were significantly enriched in lipid metabolism-related pathways. **(C)** Enriched pathway regulatory network for 26 pathways. **(D)** The gene regulatory network for the PPAR signaling pathway. **(E)** Visualization of expression of the PPAR signaling pathway (most genes were highly expressed in the mRNAsi-high subgroup). The expression of the gene in both mRNAsi subgroups ranges from -1 to 1. A value closer to 1 means that the gene is highly expressed in the high-mRNAsi subgroup (red color); conversely, it is highly expressed in the low-mRNAsi subgroup (blue color). Most of the genes in the PPAR signaling pathway are highly expressed in the high-mRNAsi subgroup. **(F)** Meanwhile, GSEA was performed to compare the high-mRNAsi and low-mRNAsi subgroups, to identify upstream pathways of PPAR signaling that were up-regulated in the mRNAsi-high subgroup, NES =2.049. PPAR signaling pathways were up-regulated in the mRNAsi-high subgroup, NES = 1.642.

To determine DEGs-enriched pathways, 845 DEGs, identified by samples’ mRNAsi (greater than median value or not), were first used to perform over-representation analysis (ORA). As shown in **Figure 3A**, the identified enriched pathways did not include the commonly reported sorafenib-associated pathway (Ras-MEK-ERK pathway) but were related to lipid metabolism. To exclude the analysis bias, more differentially expressed genes were included between the high-mRNAsi and low-mRNAsi subgroups in differential expression analysis. Then, we selected 1853 DEGs (|logFC| > 1.5, adj.P-value < 0.01) in total, to repeat pathway analysis, and similar analysis results were collected. Indeed, DEGs between the high-mRNAsi and low-mRNAsi subgroups were mainly enriched in lipid metabolism-associated biological processes, such as the PPAR signaling pathway (https://www.wikipathways.org/index.php/Pathway : WP3942) and fatty acid omega-oxidation (**Figure 3B**). It was worth noting that fatty acid biosynthesis and one of the PPAR subtypes PPARG had been reported to be associated with the efficacy of sorafenib (50, 51).

Hence, to determine the causal relationships between our enriched pathways and the response to sorafenib treatment, we first inferred regulatory relationships between the enriched pathways *via* Bayesian network (BN) inference. BN inference revealed the interactions between the current enriched pathways and other pathways. It provided a more comprehensive insight into the regulatory impact. As shown in **Figure 3C**, the most notable finding was that the PPAR signaling pathway could trigger fatty acid biosynthesis. The PPARA signaling pathway had been identified as activated in CSCs (52), and PPARA and PPARG can enhance stemness and tumorigenicity by PPAR-fatty acid oxidation program (53). We further identified the PPAR signaling pathway regulating fatty acids biosynthesis in low differentiated samples (high-mRNAsi subgroup) (**Figures 3C, E**). The results of our analysis suggested that the PPAR signaling pathway was the “bridge” between fatty acid imbalance and maintenance of cancer cell stemness.

As shown in **Figures 2D**, **3E**, notably, several genes were highly expressed in the PPAR signaling pathway. In addition, we conducted a GSEA analysis between the two mRNAsi-related subgroups using logFC. And we found that the PPAR signaling pathway and its upstream pathway nuclear receptors meta-pathway were significantly enriched in the high mRNAsi group. This result further demonstrated that the PPAR signaling pathway was a sorafenib resistance-related pathway (**Figure 3F**). This considerably different expression (NES=1.615) in two mRNAsi subgroups suggested that the PPAR signaling pathway was a sorafenib response-related pathway that deserved our attention. We hypothesized that genes involved in lipid metabolism might be related to the response to sorafenib in HCC patients and that these genes were overlooked in previous reports.

### *SCD* Is One of the Hub Genes in the PPAR Signaling Pathway That Plays a Role in Sorafenib Resistance Development

In the PPAR signaling pathway, *SCD* was reported to code for an enzyme crucial for the conversion of saturated C16/C18 fatty acids into monounsaturated fatty acids and regulation of the saturated fatty acid:monounsaturated fatty acid (SFA : MUFA) ratio. Furthermore, we inferred that the *SCD* expressed in the PPAR signaling pathway might play a vital role in sorafenib resistance. Upon visualizing the expression of the entire pathway, we found that most genes, including *SCD*, were highly expressed, compared to those in the mRNAsi-low subgroup (**Figures 2D**, **3E**).

After noting the apparent differences in *SCD* expression, we inferred the gene regulatory network in the PPAR signaling pathway and searched for *SCD* genes with regulatory functions. As shown in **Figure 3D**, upon combining literature reports (50) and regulatory networks inferred from the enriched result, we could focus on a regulatory route from *RXRB* to *NR1H3* to *CYP8B1*, and finally to *SCD*. It was well known that i) the *RXRB* TF could induce the transcription of *NR1H3 (* 54), ii) the *NR1H3* TF could induce the transcription of *SCD (* 55), and iii) the *CYP8B1* enzyme could catalyze the synthesis of lipid and cholesterol (56). Our result indicated that the high level of expression of *SCD* and its upstream genes in the PPAR signaling pathway diminished the therapeutic efficacy of sorafenib.

**Figure 4:**
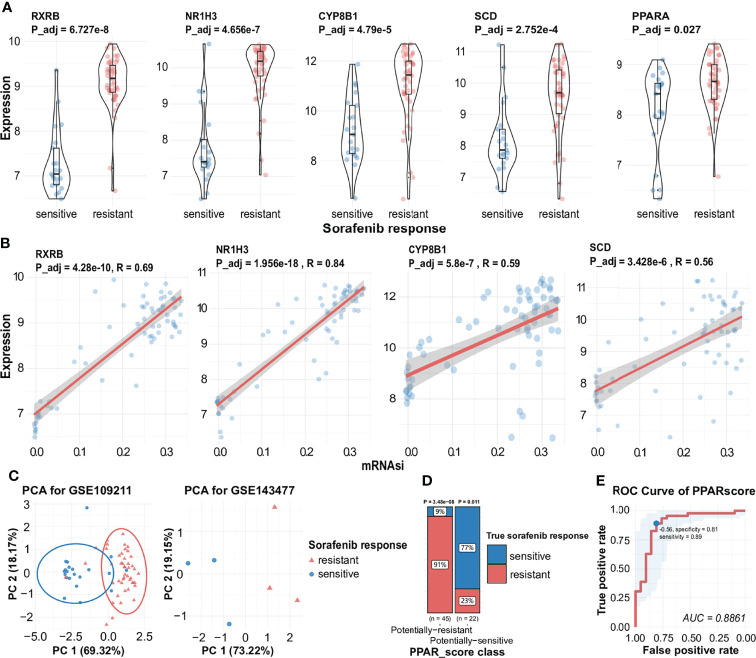
Expression of key genes during the generation of different responses to sorafenib, the correlation between key genes and mRNAsi, and visualization of PPAR-related signature. **(A)** Expression of *RXRB*, *NR1H3*, *CYP8B1 SCD*, and *PPARA* during different responses to sorafenib treatment with statistical significance. **(B)** Correlation analysis facilitated the identification of a significant positive association between mRNAsi and several sorafenib resistance genes (*RXRB*, *NR1H3*, *CYP8B1*, *SCD*). **(C)** AUC results are indicative of the expression of four genes in the sorafenib cohort GSE109211 (the left) and PCA results indicate the expression of four genes in the validated sorafenib cohort GSE143477 (the right). See also **Figure S2**. **(D)** The proportion of different responses to sorafenib (responder or non-responder) in two groups of samples for four-gene scores. **(E)** ROC of *PPARscore*, the AUC value of *PPARscore* was 0.8861. The point with the highest specificity and sensitivity in the curve was -0.56.

We further checked the correlation between related genes and their expression levels in different responses to sorafenib. Three genes (*NR1H3*, *CYP8B1*, *SCD*) were induced transcription by subtypes of PPARs (https://www.wikipathways.org/index.php/Pathway: WP3942) (57). We found that *PPARA* was slightly different expressed in two responses to sorafenib, which meant *PPARA* was responsible for the change in the expression level of its downstream target genes (**Figure 4A**, **Figure S2**). The expression levels of all the four key genes were higher in sorafenib non-responders than in sorafenib responders (**Figure 4A**). To assess the accuracy of inferred stem-related sorafenib resistance indices, we conducted correlation analysis and observed high levels of relevance between mRNAsi and the expression levels of *RXRB*, *NR1H3*, *CYP8B1*, and *SCD* (**Figure 4B**).

### The PPAR-Related Signature Can Be Used to Predict the Response to Sorafenib

To assess the predictive accuracy of the four-gene signature (*RXRB*, *NR1H3*, *CYP8B1*, and *SCD*), we used PCA to assess the ability of the signature to predict the effectiveness of sorafenib therapy, using the expression levels of the four genes. As shown in **Figure 4C**, the expression of these genes was also significantly correlated with the response to sorafenib. Hence, we calculated the four-gene signature, named *PPARscore*, for sorafenib samples using the coordinates of samples on the first 2 principal components. The Receiver-operating characteristic (ROC) analysis showed that *PPARscore* achieved an Area Under Curve (AUC) of 0.88, which *PPARscore* equaled -0.56 with the highest sensitivity and specificity (**Figure 4E**). We classified samples into two groups based on the best threshold of *PPARscore*. If the *PPARscore* was greater than -0.56, it means that the likelihood of sorafenib resistance development is higher. The PPAR-related signature was significantly correlated with sorafenib resistance (**Figure 4D**, **Table S3**). Finally, we validated *PPARscore* in another sorafenib cohort, i.e., GSE143477 (**Figure 4C**, **Table S4**). The scores of three sorafenib-resistant samples were greater than -0.56 and those of three sorafenib-sensitive samples were less than -0.56 (**Table S4**). We also examined whether the expression of the four genes showed a similar trend in the GSE143477 cohort. The expression levels of *RXRB*, *NR1H3*, and *SCD* were higher in samples exhibiting sorafenib resistance (**Figure S2**). These results suggested that the expression levels of *RXRB*, *NR1H3*, *CYP8B1*, and *SCD* in the PPAR signaling pathway were strongly associated with the response to sorafenib.

### Differences in the Expression of Key Genes are Not Related to the Somatic Mutation Frequency

**Figure 5:**
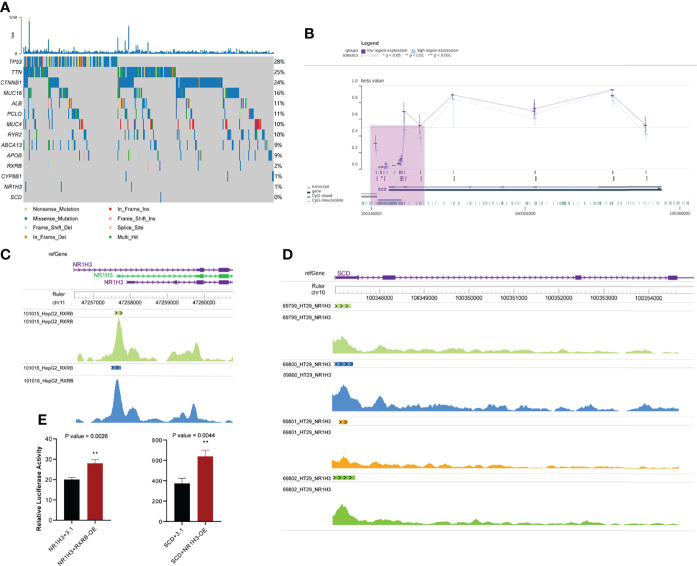
Other omics analysis of key genes. **(A)** Somatic mutations in the top 10 mutated genes and key genes in the PPAR signaling pathway. **(B)** Visualization of methylation of the *SCD* promoter region in HCC. The purple shaded area represents the *SCD* promoter region **(C)** Chips of *RXRB* bind to the promoter region of *NR1H3*. **(D)** Chips of *NR1H3* bind to the promoter region of *SCD*. **(E)** Relative dual luciferase activities of *NR1H3* and *SCD* promoters were determined at 48 h in MHCC-97h cells (Left: *NR1H3*; Right: *SCD*. pcDNA3.1-RXRB and pcDNA3.1-NR1H3 plasmid were empty pcDNA3.1 plasmids as the control). Data results were shown as mean ± SEM (n ≥ 3). P-values were calculated by two-tailed t-tests. **P<0.01; OE, overexpression; SEM, standard error of the mean.

We explored the potential regulatory mechanisms of the core genes described above. Variations in genetic expression may be attributable to the occurrence of key somatic mutations in genes within the transcriptome across patients with different phenotypes and specific types of cancer (58). We assessed the expression of key genes related to the response to sorafenib, to examine the possibility that the response to sorafenib is affected by mutations, by comparing the frequencies of somatic mutations for the top 10 mutational genes and key genes in the PPAR pathway (**Figure 5A**). We then identified key genes in the PPAR pathway that exhibited low mutation rates in the TCGA HCC dataset. This result proved that somatic mutation frequencies in key genes were not responsible for the changes in the expression of key genes. Therefore, we ruled out the possibility that mutation frequency affected the function and expression of these genes. The result mirrored the Bayesian inference that these gene-phenotype-related alterations occur mainly at the transcriptional level.

### Transcription Factors in the PPAR Signaling Pathway Induce the Expression of NR1H3 and SCD

We also assessed the potential mechanism of occurrence of alterations in gene expression, because TFs can activate gene expression by binding to the targeted gene promoter (59). We combined regulatory networks inferred from enriched results and literature reports (50, 60), and focused on a regulatory route from *RXRB* to *NR1H3* to *CYP8B1* and finally to *SCD*. Although it was well known that *RXRB* and *NR1H3* could separately bind to *NR1H3* and *SCD (* 54, 55), we demonstrated that *RXRB* and *NR1H3* could act as TFs and bind to the promoter regions of *NR1H3* and *SCD*, respectively. First, *RXRB* could bind to the promoter region of *NR1H3* in HepG2 hepatocellular carcinoma cells. Each track corresponds to a HepG2 sample. (**Figure 5C**). Second, *NR1H3* could bind to *SCD* in HT29 colorectal adenocarcinoma cells (no similar study was performed with hepatocellular carcinoma cells) (**Figure 5D**). An analysis of these results showed that there is abundant evidence to support the regulatory relationship between these genes.

Moreover, we validated whether *RXRB* and *NR1H3* could directly regulate *NR1H3* and *SCD* by luciferase reporter assay. In MHCC-97h cells, enforced *RXRB* or *NR1H3* expression significantly increased the *NR1H3* or *SCD* promoter activity. *NR1H3* or *SCD* showed transcriptional activity in response to *RXRB* or *NR1H3*. The above data demonstrated that *RXRB* or *NR1H3* can respectively bind to the *NR1H3* or *SCD* promoter and induce its transcription (**Figure 5E**).

### Similar Methylation Levels in *SCD* Promoter Regions Between Patients

Epigenetic modifications can modulate the binding of TFs to DNA; for example, DNA hypermethylation represses the binding of TFs to gene promoters (61). We checked the methylation level of the *SCD* promoter in the TCGA-HCC cohort using the MEXPRESS web server. Regardless of the level of *SCD* expression in HCC, the level of methylation in the *SCD* promoter was low (**Figure 5B**). This result suggested that the methylation of *SCD* promoter has hardly any effects on the binding of transcription factors to it. There were significant differences in the expression of *SCD* in sorafenib-resistant and sensitive groups, and *SCD* expression is probably regulated by *NR1H3*, while *NR1H3* is regulated by *RXRB*.

### Mechanistic Hypothesis Involving Four PPAR-Related Genes

**Figure 6:**
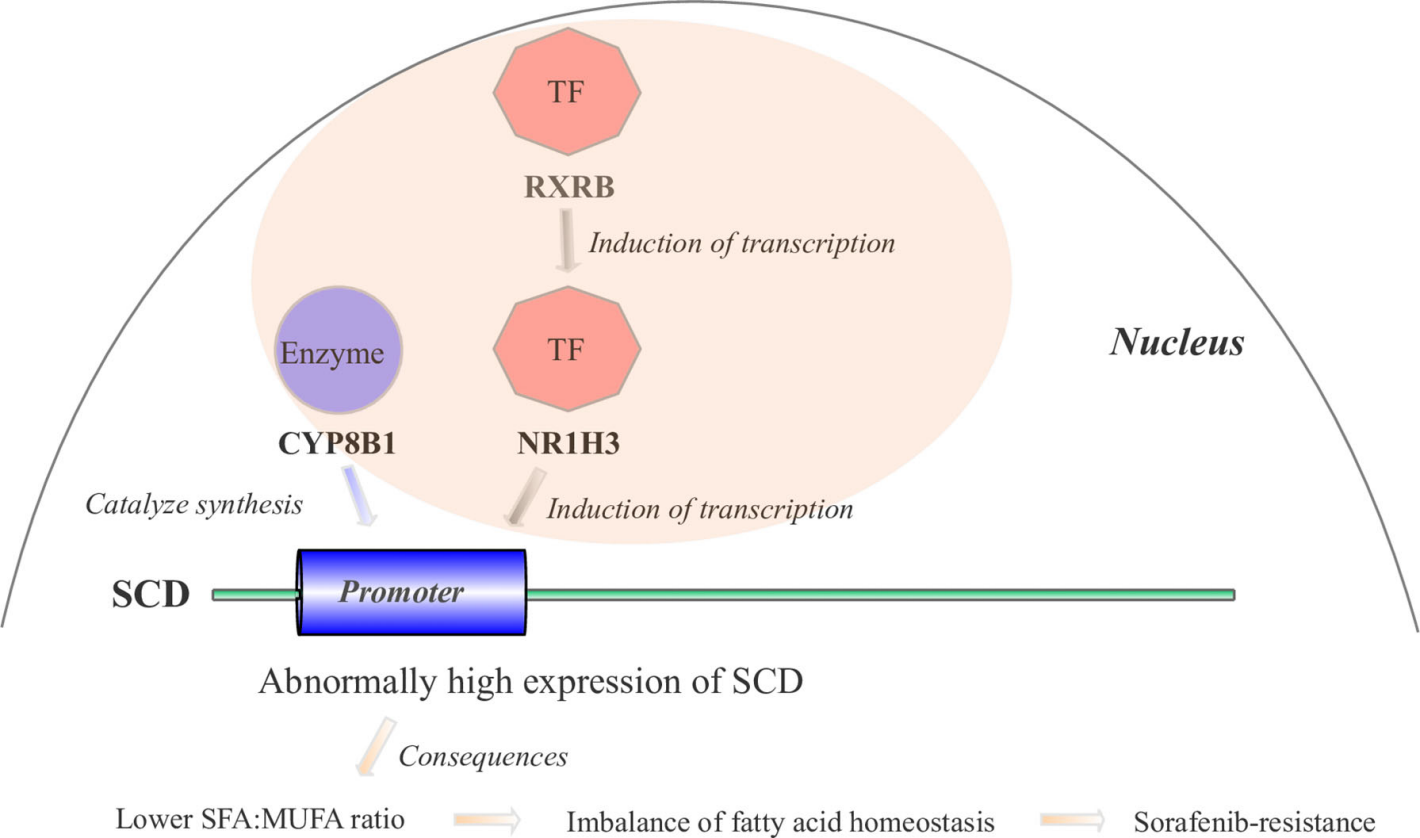
The mechanistic hypothesis for four hub genes in the PPAR signaling pathway. Abnormally high expression levels of four hub genes result in sorafenib resistance through a cascade reaction in the PPAR pathway.

Based on our BN inference results (**Figure 3D**) and literature reports, we proposed the hypothesis that four gene cascades result in sorafenib resistance. As shown in **Figure 6**, the *RXRB* TF induced the transcription of *NR1H3* and the transcription of *SCD* was induced by the TF *NR1H3* and the enzyme *CYP8B1*. The high level of expression of *SCD* results in a lower SFA : MUFA ratio, and further causes an imbalance in fatty acid homeostasis and sorafenib resistance development.

### mRNAsi Is a Valuable Prognostic Predictor for HCC Patients

**Figure 7:**
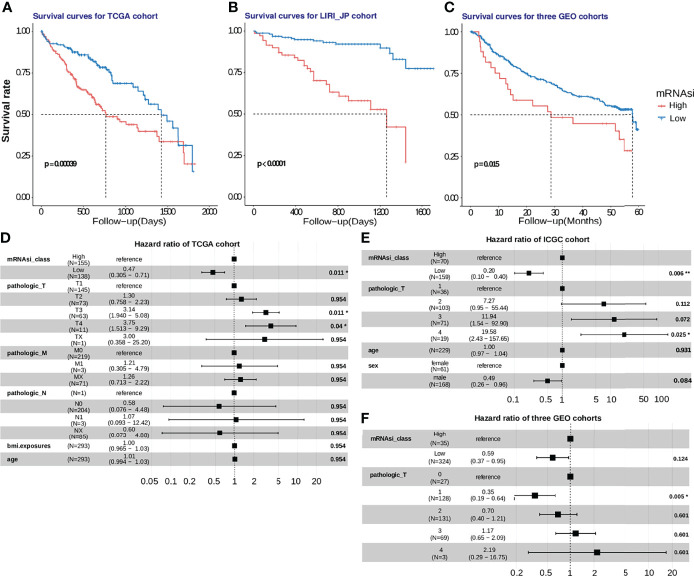
Development and validation of the mRNAsi cutoff value in five HCC cohorts. **(A)** In the TCGA-HCC cohort, patients with high stemlike indices (mRNAsi > 0.55) suffered from worse survival outcomes, compared to those in the low-mRNAsi group with log-rank test P =0.00039. **(B, C)** The mRNAsi cutoff value was further verified using the ICGC-JP cohort and 3 GEO cohorts, and the mRNAsi-based cutoff was a significant hazard factor for HCC patients with log-rank test p<0.0001 and p = 0.016. **(D, E, F)**. The hazard ratios of mRNAsi were shown using a forest plot in the training and validation cohorts.

We also calculated the mRNAsi for HCC samples obtained from five cohorts. A higher mRNAsi represents a lower level of differentiation of a sample (20). Survival analysis was performed only using samples obtained from patients for whom the survival duration was less than 5 years. Upon selecting the median mRNAsi value of 0.55 as the cut-off value in the TCGA HCC cohort, a 5-year survival analysis was performed. K-M analysis revealed that patients with a low mRNAsi had a better OS than those with a high mRNAsi (P = 0.00039; **Figure 7A**). Then, the prognostic value of mRNAsi was validated using the ICGC-JP cohort and three GEO cohorts with the same cutoff (**Figure 7B**; P < 0.0001; **Figure 7C**; P = 0.015). To examine whether the mRNAsi was independent of other clinical and pathological factors, we performed a multivariable cox proportional hazard analysis, by including individual clinical variables and mRNAsi subgroups in these datasets. As shown in **Figure 7D**, in the TCGA-HCC cohort, the mRNAsi class and the TNM Staging System (TNM) were significantly associated with the OS during multivariate analysis. The mRNAsi class was significantly associated with the OS in ICGC-JP cohorts (**Figure 7E**). But the adjusted P value of mRNAsi class is no longer significant in three GEO cohorts (**Figure 7F**). These results suggest that mRNAsi may is a robust predictive factor of HCC patient survival.

## Discussion

HCC is the most common primary liver cancer in adults and is the leading cause of cancer-related mortality worldwide (62, 63). Sorafenib is the only first-line chemotherapeutic treatment administered to advanced HCC patients (5, 6). Sorafenib therapy has proven to be effective in the treatment of patients with advanced HCC. Given that the overall rate of response to sorafenib therapy is still low (1, 7), it is crucial to identify patients who can benefit the most from sorafenib therapy. Here, the predictive value of the stemness index for response to sorafenib treatment was first confirmed. Despite evaluating different markers in sorafenib-resistant HCC for several years, we did not discover a promising index that could predict the response to sorafenib therapy. This highlights the need to identify a biomarker for sorafenib treatment in HCC. By applying mRNAsi to sorafenib therapy cohorts, novel mRNAsi-based subgroups that could enable us to understand the response to sorafenib were clustered. Two mRNAsi-based subgroups were strongly correlated with the sensitivity to sorafenib therapy. Hence, we performed a functional enrichment analysis of DEGs, to determine whether common sorafenib-related pathways were differentially expressed in these two subgroups. We also assessed the effect of several selected DEGs on the results of enrichment analysis (845 DEGs, followed by 1853 DEGs, along with 845 genes included in the unsupervised cluster have been shown in **Table S2**). Hence, in order to further clarify the relationship between enriched pathways, we performed Bayesian network inference using *CBNplot*, which helped us to identify the previously overlooked regulatory relationship between pathways. It was inferred that the PPAR signaling pathway regulated fatty acid biosynthesis. This suggested that the change in lipid metabolism might be related to the response to sorafenib in HCC patients, but had been overlooked in previous reports.

It is known that fatty acids (FAs) can be broadly classified as saturated FAs and unsaturated FAs. Different ratios of unsaturated to saturated fatty acids (UFA: SFA ratio) can affect tumor cell survival, as high levels of saturated fatty acids result in lethal lipotoxicity (50). However, unsaturated fatty acids cannot induce reactions to metabolic stress and thus repress lipotoxicity (55). The PPAR signaling pathway can mediate not only saturated fatty acid synthesis, but also monounsaturated fatty acid synthesis (64). Stearoyl-coenzyme A desaturase 1 (*SCD*) can convert saturated fatty acids into monounsaturated fatty acids in the PPAR signaling pathway (65). The role of *SCD* in facilitating hepatocarcinoma cell proliferation and efficacy of sorafenib treatment has been confirmed (50, 66). We identified that the PPAR pathway regulating fatty acid biosynthesis also affects the efficacy of treatment with sorafenib. Recent studies have verified that PPAR can enhance stemness and tumorigenicity in individuals consuming a high-fat diet (53, 67). This explained why the PPAR signaling pathway is strongly associated with mRNAsi stemness indices and sorafenib resistance. It has been demonstrated that the inhibition of PPARG, one subtype of the peroxisome proliferator-activated receptor, could reverse the metabolic reprogramming of compensatory glutamine and further sensitize HCC cells to sorafenib (51). This implies that the PPAR signaling pathway plays an important role not only in glutamine metabolism but also in fatty acid homeostasis. In addition, we demonstrated that the different levels of expression of PPAR-related genes were correlated with sorafenib resistance, and were not attributable to somatic gene mutations. The rate of occurrence of somatic mutations in resistance-related genes was almost zero. It has also been demonstrated that TFs such as *RXRB* and *NR1H3* can bind to the promoter regions of their target genes (*NR1H3*, *SCD*). We also verified that the methylation level of the SCD promoter hardly affected its binding with NR1H3. Finally, we proposed the hypothesis that key genes cascades result in sorafenib resistance, based on literature reports and gene regulatory networks. The PPAR signaling pathway is activated in samples with obvious stemness feature (higher stemness indices). Four genes, *RXRB*, *NR1H3*, *CYP8B1* and *SCD*, were involved in PPAR signaling pathway (57) act as sorafenib-resistant related genes. It is known to *RXRB* induce the transcription of *NR1H3 (* 54). And the transcription of *SCD* was induced by the TF *NR1H3 (* 55). The *CYP8B1*, as a catalyze enzyme, induced lipogenesis, whose overexpression increased *SCD* expression (68, 69). *NR1H3* as TF and *CYP8B1* as catalyzing enzyme induce *SCD* expression. Finally, increased expression of *SCD* results in greater content of MUFA and lower SFA : MUFA ratio, causing an imbalance in fatty acid homeostasis (50, 55). The imbalance in fatty acid homeostasis subsequently increased and maintain the stemness of cancer cells and further resulted in sorafenib resistance development (50, 70).

The prognosis of individual patients varies greatly due to high levels of heterogeneity (62). Hence, we need to urgently develop novel diagnostic or prognostic biomarkers that can predict multiple HCC cohorts. Accumulating evidence demonstrates that mRNAsi can predict the prognosis of other cancers (71, 72). Based on this, we employed a trained one-class regression model, and scored the mRNAsi for each of the HCC patients, by determining the Spearman correlation between the weight vectors of the stemness signature, mRNA expression data, and the mRNAsi threshold, to predict a better or worse prognosis, and set it at 0.55. Survival analysis was performed using data of patients with HCC from the TCGA cohort and validated with data from the ICGC-JP cohort and three GEO cohorts. K-M analysis demonstrated the effective stratification of low- and high-risk patients according to different results for overall survival, suggesting that the stemness index could be used as a robust prognostic marker. Multivariate cox regression analysis suggested that the prognostic capacity of the stemness likeness was independent of other clinical data. In general, the lower mRNAsi score represented a better survival prognosis, and an mRNAsi value of 0.55 can be used as a cutoff for predicting the prognosis of HCC patients, which was validated in both the TCGA cohort and the four independent datasets.

However, several limitations were associated with our work, and need to be optimized in the future. The validation of other omics was different from that of the HCC cohort used to identify mRNAsi-related subgroups.

In this study, we performed a systematic analysis of the mRNAsi subgroups that were strongly correlated with the response to sorafenib. Simultaneously, HCC stem-like indices that were based on multiple independent cohorts were used to validate the robust prognostic ability of mRNAsi. To our knowledge, this is the first attempt to explore the potential mechanisms of the development of sorafenib drug resistance by assessing the tumor stemness likeness. Through an analysis of differentially expressed pathways between two mRNAsi-related subgroups in sorafenib cohorts, we identified the PPAR signaling pathway to be associated with sorafenib therapy. The key genes *RXRB*, *NR1H3*, *CYP8B1*, and *SCD* were identified in the PPAR signaling pathway, and their regulatory relationships were also examined. They can be used as candidate targets for researching drug resistance mechanisms. In particular, *SCD* has been experimentally validated to be responsible for sorafenib resistance (50, 66). We also derived the four-gene signature that would enable us to predict the effectiveness of sorafenib therapy and formulated a mechanistic hypothesis for the four PPAR-related genes. Based on the results of our study, we thought that, in addition to commonly reported pathways, PPAR-related activities associated with fatty acid metabolism might also affect the response to sorafenib treatment. Furthermore, based on a combination of experimental evidence derived from previously conducted research (50, 66), we suggested that the combined use of SCD inhibitors and sorafenib may be a promising therapeutic approach that could be used in the future.

## Data Availability Statement

The original contributions presented in the study are included in the article/**Supplementary Material**. Further inquiries can be directed to the corresponding authors.

## Author Contributions

TF, TW, YZ, and LZ (4th Author) were the major contributors who made substantial contributions to the conception, data collection, and manuscript writing of this project. SL, ML, EH, QW, XF, and LZ (11th Author) provided crucial technical support to this study. LL, ZX, and WX provide support in data visualization for this study. XH, XS, and GY supervised this study, provided support in all aspects throughout the progression of this study, and approved the final version of the manuscript.

## Conflict of Interest

The authors declare that the research was conducted in the absence of any commercial or financial relationships that could be construed as a potential conflict of interest.

## Publisher’s Note

All claims expressed in this article are solely those of the authors and do not necessarily represent those of their affiliated organizations, or those of the publisher, the editors and the reviewers. Any product that may be evaluated in this article, or claim that may be made by its manufacturer, is not guaranteed or endorsed by the publisher.
