# Supplementary material for: Stemness Analysis Uncovers That The Peroxisome Proliferator-Activated Receptor Signaling Pathway Can Mediate Fatty Acid Homeostasis In Sorafenib-Resistant Hepatocellular Carcinoma Cells
Source: Front Oncol. 2022 Jul 22;12:912694. doi: 10.3389/fonc.2022.912694 (PMC9361019; doi:10.3389/fonc.2022.912694)
Supplement: Supplementary file 1 [file DataSheet_1.docx]

Supplemental Information
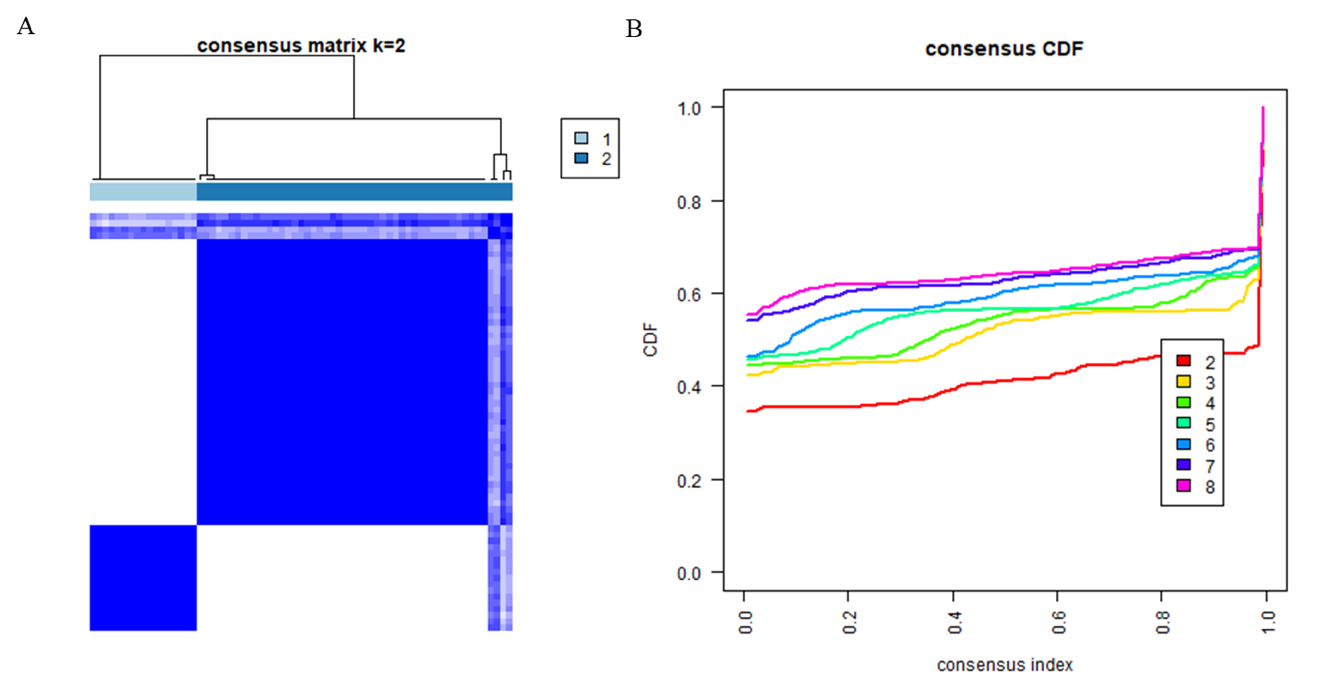


**Figure S1: Consensus matrix of sample cluster and CDF area in GSE109211**

(A) Consensus matrix of samples in two clusters. (B) CDF area of cluster numbers of 2 to 8 (choosing a curve with less fluctuation). The ConsensusClusterPlus package was utilized to distinguish stemness molecular subgroups of the response of sorafenib, and we iterated 1000 times for the optimization and stabilization of classification categories. And we chose K = 2 (two clusters: distinct consensus matrix of the cluster with much white and little light blue; CDF curve with less fluctuation).


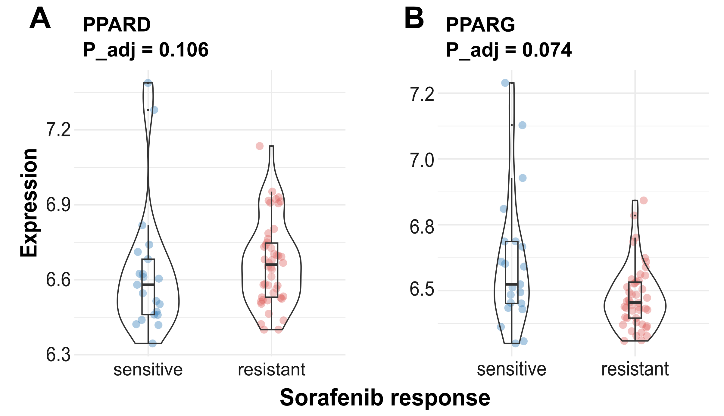


**Figure S2: Expression of 4 genes related to PPARs in different responses of sorafenib treatment in GSE109211.**

(A, B) The expression of two subtypes of PPARs (PPARD and PPARG) were not significantly different.


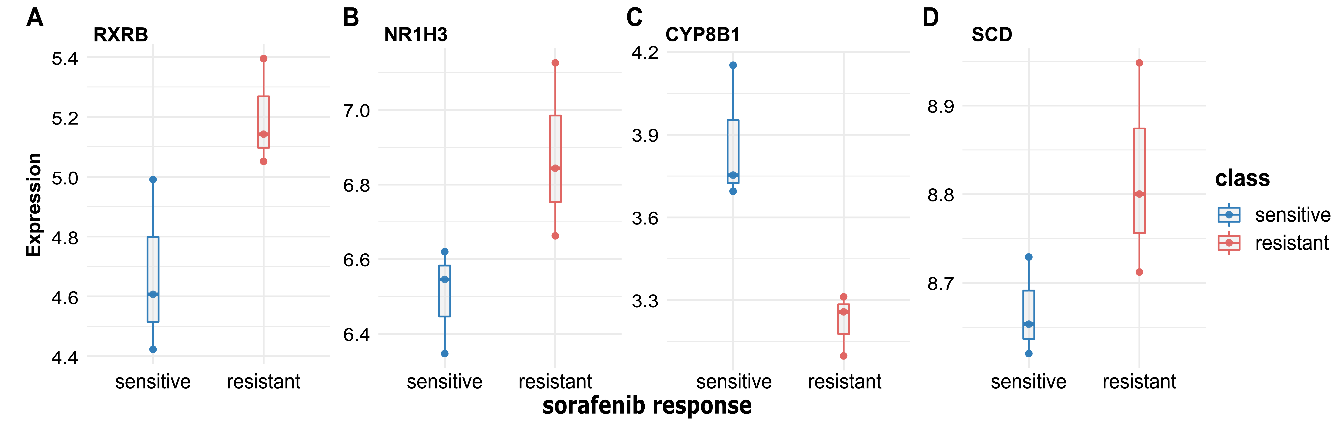


**Figure S3: Expression of 4 genes in different responses of sorafenib treatment in GSE143477.**

(A, B, D) Expressions of RXRB, NR1H3, and SCD were higher in three sorafenib-resistance samples. (C) But the expression of CYP8B1 was not as expected.

**Table S4: Result of *PPARscore* for GSE143477**

|  | Dim.1 | Dim.2 | PPARscore | ture_response | PPARscore_class |
| --- | --- | --- | --- | --- | --- |
| HepG2_NC_1_NS | 1.089 | -0.337 | 0.752 | resistant | resistant |
| HepG2_NC_2_NS | 2.331 | -0.630 | 1.701 | resistant | resistant |
| HepG2_NC_3_NS | 1.311 | 1.581 | 2.892 | resistant | resistant |
| HepG2_sh_sox9_1_NS | -1.408 | 0.324 | -1.084 | sensitive | sensitive |
| HepG2_sh_sox9_2_NS | -2.591 | 0.252 | -2.340 | sensitive | sensitive |
| HepG2_sh_sox9_3_NS | -0.731 | -1.190 | -1.921 | sensitive | sensitive |
